# Clinical and pathologic factors predicting reclassification in active surveillance cohorts

**DOI:** 10.1590/S1677-5538.IBJU.2017.0320

**Published:** 2018

**Authors:** Pablo S. Sierra, Shivashankar Damodaran, David Jarrard

**Affiliations:** 1 Fundacion Valle del Lili -Universidad Icesi, Cali, Colombia; 2 Department of Urology, University of Wisconsin School of Medicine and Public Health, Madison, WI, USA; 3 University of Wisconsin Carbone Cancer Center, Madison, WI, USA

**Keywords:** Prostatic Neoplasms, Neoplasm Grading

## Abstract

The incidence of small, lower risk well-differentiated prostate cancer is increasing and almost half of the patients with this diagnosis are candidates for initial conservative management in an attempt to avoid overtreatment and morbidity associated with surgery or radiation. A proportion of patients labeled as low risk, candidates for Active Surveillance (AS), harbor aggressive disease and would benefit from definitive treatment. The focus of this review is to identify clinicopathologic features that may help identify these less optimal AS candidates.

A systematic Medline/PubMed Review was performed in January 2017 according to PRISMA guidelines; 83 articles were selected for full text review according to their relevance and after applying limits described.

For patients meeting AS criteria including Gleason Score 6, several factors can assist in predicting those patients that are at higher risk for reclassification including higher PSA density, bilateral cancer, African American race, small prostate volume and low testosterone. Nomograms combining these features improve risk stratification.

Clinical and pathologic features provide a significant amount of information for risk stratification (>70%) for patients considering active surveillance. Higher risk patient subgroups can benefit from further evaluation or consideration of treatment. Recommendations will continue to evolve as data from longer term AS cohorts matures.

## INTRODUCTION

Currently, 15-20% of men are diagnosed with prostate cancer (PC) during their lifetime, but the mortality risk is only 3% (1). The incidence of small, low risk, well-differentiated PC is increasing, mainly as a result of PSA screening and increased intensity prostate biopsy schemes (2). Many of these patients would never develop symptomatic disease during their lifespan and therefore do not benefit from definitive treatment due to the indolent behavior of low risk PC (3, 4). It is estimated that 45% of PC patients diagnosed with PSA screening are candidates for conservative treatment and about 2/3 of these men will ultimately avoid definitive treatment (4, 5). The utilization of AS is increasing and in a recent analysis utilizing the CAPSURE database, the use of AS ranged from 6.7% to 14.3% from 1990 through 2009 but increased sharply between 2010 to 2013 when 40.4% of low risk patients had opted for this approach (6).

AS is an accepted option, and one of first line treatments offered for the initial management of low risk PC patients. Low-risk PC has been defined by D'Amico as Gleason score of 6 or less, PSA less than 10 mg/mL, and a tumor that is either non-palpable or only palpable in less than half of one lobe of the prostate (clinical stage T1c or T2a) (7). These patients have a low possibility of reclassification with only 16-18% of men requiring treatment at 2 years in several large AS cohorts (8-11). The main objective for treatment planning is trying to achieve the correct timing of curative treatment once previously established thresholds that indicate aggressive disease (disease progression) are reached, in order to preserve quality of life and avoid risk of complications associated with other definitive therapies, including surgery and radiation.

It is expected that up to 30-50% of patients with low risk disease would be upgraded to Gleason score (7 or greater) or upstaged at the time of radical prostatectomy (RP) (12-14). This is clinically important, because Gleason score upgrading including pattern 4 results in worse oncologic outcomes such as increased risk of biochemical recurrence, need for adjuvant therapy and increase of cancer-specific mortality (15). A recent longer-term analysis of an AS cohort de-monstrated that patients with GS4 have a higher risk of metastasis (16). It is imperative to define patients at low risk of progression after diagnosis and differentiate them from patients with occult aggressive cancer behavior. It is also important to obtain patient progression data from actual AS cohorts, rather than from radical prostatectomy specimens, which will require waiting additional years for current larger cohorts to mature because of the long disease history.

## MATERIALS AND METHODS

A systematic MEDLINE/PubMed review was performed in mid 2016 according to the PRISMA guidelines. The main search terms were “active surveillance”, “prostate cancer” and “upgrading” as a secondary keyword, 2168 articles were retrieved, 1599 articles remained after applying limits for papers published after 2008. The search result was first screened for relevance by title and limited to English and Spanish literature, abstracts were read and 83 publications of interest were selected for full text review. The search was complemented with own files articles, hand search and articles suggested by important publications until no additional papers were retrieved. The search sensitivity was evaluated by comparing the articles identified against those already known by the authors and those cited in previous seminal reviews. If an article was missed, the title and abstract where carefully reviewed to identify terms that would improve the sensitivity.

## OPTIMAL CANDIDATES FOR ACTIVE SURVEILLANCE

The selection of men for AS has been traditionally guided by clinicopathological features that indicate the patient presents with an organ confined, well-differentiated tumor. Life expectancy and comorbidities are important determinants for a watchful waiting (WW) versus AS strategy. Patients with a limited life expectancy are better candidates for WW which consists of less frequent PSA monitoring, no further prostate biopsies, and the application of androgen deprivation therapy if the disease becomes metastatic. For patients on AS, this switch to WW does not occur infrequently. Stratified by age, 10% of 55-year-old men switched to WW, but 50% of 70-year-old men moved to watchful waiting in one study (17). In WW, a strategy of non-invasive monitoring with PSA and DRE every 6 months leads to very low prostate cancer-specific mortality at 10 years, making this an effective strategy in carefully selected patients (18).

Current available information is limited to non-randomized clinical trials with follow-up of less than 10 years (the majority <5 years) and the recommendation from major clinical guidelines is that AS should be offered only in selected lower risk patients. However, there is no consensus on the optimal method of selecting men for AS, and depending on the clinical guidelines or on studies from different cohorts, selection criteria can change. The National Comprehensive Cancer Network (NCCN) guidelines define very low risk PC as those with T1c disease, Gleason score ≤6, PSA <10ng/mL, fewer than three positive biopsy cores, with ≤50 percent cancer in each core, and a prostate-specific antigen density (PSAD) <0.15ng/mL/g. In this very low-risk group, the NCCN recommends active surveillance as the preferred option for those with a life expectancy less than 20 years. Very low risk patients in the Epstein criteria employ one or two positive cores, no core with more than 50% involvement and a PSA density of less than 0.15. Epstein low risk disease is defined as Gleason T1- T2a disease along with Gleason score ≤6 and PSA <10ng/ mL. In this category, NCCN recommends AS, surgery or radiation if life expectancy >10years (19). The American Urological Association (AUA) (20), and The European Association of Urology (EAU) (21) recommendations are listed in Table-1 with other commonly used criteria.

**Table 1:** Commonly utilized clinicopathologic criteria for active surveillance.

AS criteria	PSA	Clinical stage	Gleason	Core	PSAD	Age/Life expectancy
NCCN very low risk	10	T1c	6	<3 core / <50%	< 0.15	<20 years
NCCN low risk	10	T1- T2a	6			>10 years
CCO			6	Low volume		
			7(3+4)	<10 - 20% of 4 pattern		>75 years
AUA	10	T1c - T2a	6			
EAU	10	T1c - T2a	6	< 3 core / < 50%	< 0.15	>10 years

**NCCN** = National Comprehensive Cancer Network; **CCO** = Cancer Care Ontario; **AUA** = American Urological Association; **EAU** = European Association of Urology; **PSAD** = PSA density

One concern is limiting this population so severely that relatively few patients would be candidates. Cancer Care Ontario (CCO) guidelines, in addition, recommends GS 7 cancer [3+4], who have low-volume and percentage Gleason 4 pattern pathology (<10 to 20 percent pattern 4), and/or age older than 75 years (22). (Table-1).

Overholser et al. compared outcomes between men with very low risk (Epstein criteria) and low-intermediate risk disease (4 or fewer cores with GS 6 cancer and/or only 1 core of Gleason 3+4 cancer <15%) and found no differences in adverse pathological features such as upgrading or upstaging at radical prostatectomy (23). There was an obvious increase in patients eligible for AS from 44% to 68% of their population by expanding these selection criteria. The risks of adverse specific outcomes will likely be higher with the inclusion of men with more in-termediate risk features, but the absolute risk of death may still be low (24). In the previously described study (23), only two of the 320 patients either experienced metastatic disease or died of PC during the 12-year study period. Notably, in the recent TGCA analysis, GS 6 tumors were all diploid, but increased grade greater than 4 was associated with increased polyploidy (25). The new International Society of Uropathology (ISUP) Gleason grade groups classifies Gleason 6, 3+4, 4+3, 8 and 9-10 as grade groups of 1, 2, 3, 4 and 5 respectively (26). The adaptation of this grade system by urologists will witness incorporation of this system in active surveillance protocols.

Expanding criteria for AS without compromising absolute outcomes will be provided in the future with additional follow up in surveillance cohorts.

Mortality and quality of life are indicators that have been infrequently applied to these patient populations and will be an area of development in the future. Bellardita et al. summarized the evidence on quality of life in AS cohorts in a systematic review in 2014 examining ten research based studies that measured this outcome (27). Patients undergoing AS reported good quality of life in diverse questionnaires and did not appear to suffer major psychological impact, indeed overall scores were comparable or better than those of patients undergoing radical treatment.

Another factor associated with AS is the risk associated with monitoring itself, namely infection or other complications after prostate biopsy (28). A large population based study demonstrated that patients undergoing a transrectal biopsy had more than twice the risk of hospitalization within 30 days of the procedure compared to a control population (29). There is a recent chronological trend towards increased infection with antibiotic resistant organisms after transrectal biopsy (30, 31). Bleeding and pain from the procedure also impact patients undergoing prostate biopsy. Hematuria and hematospermia are common after the procedure, but typically mild and self-limiting (32).

Selection criteria and patient populations continue to evolve because AS cohorts still have relatively short follow-up compared to PC's natural evolution.

## CLINICAL AND PATHOLOGIC FACTORS THAT PREDICT RECLASSIFICATION

There is a concern in clinical practice that some patients diagnosed initially as low-risk PC actually have aggressive disease. Many studies have demonstrated the frequent disparity between Gleason scores reported on prostate biopsy and at radical prostatectomy, that is in part explained because of misdiagnosis on first biopsy or also because most AS programs only use Gleason score, clinical stage and PSA to enroll patients. excluding other possible important predictors such as age, PSA density (PSAD), prostate volume, obesity, and race. These features are summarized in Table-2.

**Table 2:** Clinicopathologic predictors of upgrading gleason score (>6) during active surveillance from published studies.

Markers	Studies	Correlates	Strength
No. positive cores	Bul et al., Klotz et al., Truong et al., Iremashvili et al.	Higher cancer volume	+++
Max. % core involvement	Klotz et al., Truong et al.	Higher cancer volume	+++
PSA Density	Bul et al., Kobt et al., San Francisco et al., Truong et al., Iremashvili et al., REDEEM	Higher cancer volume, Low testosterone	+++
Small prostate volume	Freedland et al., Davies et al., Gershman et al.	Low testosterone	++
Obesity	De Cobelli et al., Truong et al.	Low testosterone, hyperestrogenism, hyperinsulinemia, elevated adipokines	++
Inherited cancer syndromes	Castro et al., Bratt et al.	Aggressive cancer, metastases risk	+
Hypogonadism	Pichon et al., Gao et al.	Aggressive cancer	+
African American ethnicity	Rebbeck et al., Sundi et al., Abern et al.	Aggressive cancer, advanced stage and poorer outcomes	+
PSA velocity	Kates et al.	Aggressive cancer/ >4 years on surveillance	+/-
Family history	Hemminki et al., Kupelian et al., Kundu et al., Selkirk et al..	Aggressive cancer	+/-

**+++** = Strong association; **++** = Moderate association; **+** = Weak association; **+/**- = Doubtful association

Radical prostatectomy specimens provide an opportunity to determine the actual grade and stage of low risk patients in modern cohorts and determine those features that best predict upgrading. Further pretreatment risk stratification in patients labeled as “low risk” is of paramount importance. There are externally validated clinical predictors (nomograms) that could help identifying this population (33). Delaying definitive treatment in this kind of patients could result in disease progression and adverse outcomes (34, 35).

### Tumor Volume

In most malignancies, tumor volume is an important indicator for disease aggressiveness as well as in PC, but one unique aspect of PC is its multifocal origin (36). The physician usually has only an indirect estimation of tumor volume in the TRUS biopsy based on the number of cancerous cores, and the percentage of cancer involvement within each core. Unfortunately, at TRUS biopsy, the current standard for PC diagnosis, larger prostates may be under sampled or the tumor volume underrepresented.

Even with the above caveats that TRUS biopsy has, indirect representation of tumor volume by assessing maximal core involvement and number of cores with cancer has been found to be important predictors for GSU, AS failure and functions as a trigger that indicates the necessity of treatment of these patients. Bul et al. found increasing number of positive cores as one of the strongest predictors for reclassification (OR 2.2; 95%CI 1.67-2.81; p<0.001) (8). Klotz et al. described number of cores involved with cancer as a factor that correlates with risk of reclassification in the largest AS cohort described to date (10). Results are corroborated by other investigations (37, 38).

Higher tumor volume has a direct proportional relationship with probability of reclassification on follow-up biopsies and subsequent AS failure. Furthermore, having bilateral tumor in the initial biopsy or developing a bilateral tumor on follow-up biopsies are associated with an increased risk of failing AS because of increasing cancer cores >2, increasing maximal core involvement >50% and Gleason score upgrading (HR 3.8; 95% CI 2.046-7.058; p<0.0001). This association was encountered in our AS cohort [unpublished data].

### PSA density

Major AS cohorts and studies have repeatedly demonstrated PSA density (PSAD) can predict progression of the disease. The multinational European study PRIAS found PSAD to be one of the two strongest predictors for reclassification and switching to definitive treatment (8). Kobt et al. recognized in their AS cohort that PSAD‹ 0.15 may suggest indolent disease (39) and San Francisco et al. went further and reported that a PSAD greater than 0.08 can predict pathological progression (40). These results were confirmed recently in a larger AS cohort (41). PSA density seems to be a key factor in predicting PC progression, in the first sub analysis of the REDEEM study the authors could not prove dutasteride prevents progression in AS, but found PSAD to be a predictor for disease progression (42). It appears that PSAD should be included not only as a selection criterion but also as a trigger for intervention or as an indicator of AS failure (OR 3.0 (2.14 – 4.28, p<0.01) (8). Smaller prostates are more prone to GSU at radical prostatectomy possibly because a small gland size may be a marker of lower androgenicity, and cancer growing in this environment may have a more aggressive biology as suggested previously (43). Several studies have suggested that lower volume prostates harbor a greater potential for cancer upgrading. Davies et al. and Gershman et al. found a smaller prostate size to be an independent predictor of upgrading OR 0.58 and OR 0.59 respectively, and these results have been corroborated in different publications (44, 45).

Most major urological societies recommend PSAD as a selection criterion in clinical guidelines and AS cohorts (being adopted now in the NCCN guidelines, PRIAS study (8), Johns Hopkins cohort (11) because previous studies not using PSAD as a cutpoint have demonstrated higher rates of upgrading in AS populations (10, 33).

### Obesity

Increased body mass index as a component of the metabolic syndrome is a major risk factor for deleterious chronic diseases like diabetes, cardiovascular disease, hypertension and stroke. Obesity generates high levels of insulin and IGF-1, elevated estrogen, reduced testosterone and increased levels of adipokines that create an environment of chronic subclinical inflammation that would favor the development of more aggressive PC (46). Compared with normal or overweight men eligible for AS, obese patients are at higher risk of upgrading or upstaging. Furthermore, obesity (BMI>32) has been found to be an independent predictor of upgrading of low risk patient biopsies (odds ratio of 1.90) as one of the four criteria assessed in the validated BADGR nomogram (37).

### Other Clinical predictors

Other less powerful clinical predictors for reclassification include low serum testosterone and race. Pichon et al. in a cohort taken to radical prostatectomy found GSU from predominant Gleason score 3 to predominant Gleason 4 more commonly in hypogonadal patients (total testos-terone <3) when compared to patients with normal testosterone (20.1% vs 11.6% P=0.002) (47). In a more recent trial measured serum testosterone in 167 low risk PC patients eligible for AS that underwent RP revealed preoperative testosterone was lower in the upgraded compared to the non-upgraded group (3.72 vs 4.56, P<0.01) (48).

African American (AA) men have a higher incidence of PC compared to other racial groups, and present with more advanced stages at diagnosis, more aggressive tumors and poorer outcomes (49). It is still uncertain if race is a prognostic factor for upgrading in AS cohorts. African American men with very low risk PC who met the criteria for AS but undergo RP experience significantly higher rates of upgrading and adverse pathology compared to other races (27.3 vs 14.4 P<0.001) (50) suggesting concern for AS in this cohort. However, major AS cohorts underrepresent minorities, only 6% to 10% of those men are African American, thus the oncologic outcomes of this particular subgroup of patients is unknown. In an effort to examine results from AS cohorts, Iremashvili et al. (38) analyzed a cohort of 24 AA in AS and found an adjusted hazard ratio of 3.8 for reclassification on serial biopsy. In a large multiethnic cohort with 39 African American men on AS (Duke Prostate Centre), black race remained the sole predictor of treatment (HR 3.08, P = 0.01) (51). Sundi et al. (52) also reported a significant higher rate of upgrading of AA compared to Caucasian men (32.7% vs 12.6% p<0.001). Further study with larger populations, and subtyping of different AA ethnicities is required before a definitive conclusion can be reached regarding this issue.

Having a first-degree family member with PC increases the risk of PC diagnosis (53). Family history is a well-known risk factor for PC, but literature is scarce and contradictory on whether it is a factor that predicts reclassification and AS failure. Some authors suggest that men with family history of PC have more aggressive tumors compared to patients with sporadic neoplasias (54), but more recent studies have found no difference in tumor features and reclassification among patients with or without PC background in their families (55, 56).

Cancer susceptibility genes (BRCA1-BR-CA2) represent a special subgroup in whom there is a clear trend toward more aggressive tumors. These mutations are suspected in patients with strong family history of early breast, prostate and ovarian cancer. Carriers of this mutation are at higher risk to develop more aggressive disease and higher risk of developing metastatic disease. Recent work suggests that this subgroup of patients should not be considered for AS (57, 58).

Iremashvili et al. (59) analyzed the usefulness of PSA kinetics for surveillance of patients on AS. They followed up 314 patients on AS and analyzed PSA and its derivatives namely PSA density, PSA velocity, PSA doubling time and correlated them with the risk of progression on surveillance biopsies, first through fourth. They found that PSA velocity and PSA density were associated with the risk of progression only during the fourth biopsy and hence need a minimum follow-up of 4 years to be an efficient prediction tool. Kates et al. (60) have observed that using the PC Research International Active Surveillance (PRIAS) protocol, which uses PSA kinetics along with scheduled biopsies at 1, 4 and 7 years compared to the John Hopkins Hospital (JHH) protocol of doing an annual biopsy alone in their AS cohort of 1125 men would increase the chance of an intervention by 12%, thus questioning the validity of PSA kinetics in this setting. Therefore, PSA velocity appears less useful in the short term for predicting risk of failure.

## NOMOGRAMS

Multiple prognostic models have been developed to calculate the probability of pathologic features in the prostatectomy specimen such as low grade, low volume or organ-confined disease (35, 37, 61-63), but many nomograms have demonstrated low predictive accuracy and poor ca-libration upon external validation (Table-3). The utility of the nomograms to predict insignificant disease has been assessed in several studies. Iremashvili et al. evaluated and compared the ability of 4 nomograms (Capitanio et al., Chun et al., Kulkarni et al. and Moussa et al.) to predict GSU and found that all the nomograms were poorly calibrated and not ready for clinical application (63). Wong et al. revisited other four prognostic models and reported only a moderate range of predictive ability for the Kattan et al. model (64).

**Table 3:** nomograms that predict pathologic (Gleason >6) upgrading at the time of radical prostatectomy and validation in as cohort.

Nomogram (author)	Patients (no)	Primary outcome	Variables used for formulation of nomogram	Predictive accuracy rrp (auc)	Accuracy in as* (c-index)
**BADGR (Truong)**	413	Probability of Gleason upgrading	PSAD, BMI, # Positive cores, Max % cancer core involvement	0.753	0.671
**Kulkarni**	175	Probability of Gleason upgrading	PSA, Age, Pathologist , DRE, PIN, TRUS volume, Hypoechoic lesions on TRUS, Type of biopsy, % Cancer in biopsy	0.71	0.609
**Dinh**	5,581	Probability of Gleason upgrading	Age, PSA, % positive cores	NA	0.560
**Kattan**	409	Probability of indolent tumors	PSA, Clinical Stage, Primary Gleason, Secondary Gleason, TRUS volume, Length of cancer & benign tissue in the biopsy material	0.79	0.687
**Partin**	5,629	Probability of pathologically non organ confined disease	PSA, Clinical stage, Gleason score	0.702	0.537

**PSA** = Prostate specific antigen; **PC** = Prostate Cancer; **TRUS** = transrectal ultrasound; **PSAD** = Prostate specific antigen density; **BMI** = Body mass index; **DRE** = Digital rectal examination; **PIN** = Prostate intraepithelial neoplasia

* Harrel’s C- Index based on “Predictive models and risk of biopsy progression in active surveillance patients” by Iremashvili et al. (63)

More recently, the performance of different radical prostatectomy-based prognostic tools in predicting biopsy progression were analyzed (Partin et al., Dinh et al., Kattan et al., Truong et al. and Kulkarni et al.) in AS patients and results indicate that the Kattan and Truong (BADGR) nomograms had a higher predictive value than the other tools and were able also to stratify patients into subgroups with different risk of progression at the time of diagnosis (65). These two nomograms have important differences, which suggest that a more effective predicting model combining the strong sides of both tools could be developed (Table-3).

Truong et al. assessed more than 30 clini-copathological parameters and using multivariable logistic regression found PSAD, obesity, number of positive cores and maximum core involvement (BADGR nomogram) to be useful for predicting upgrading (Gleason ≥7) on final pathology. An online risk calculator has been provided <https://www.urology.wisc.edu/research/researcherslabs/jarrard/prostatecancerpredictor> (66). The BADGR and Kattan nomograms are very similar, the main difference between them is the inclusion of BMI and PSAD in the former. Notably, the BADGR nomogram is designed to predict pathological upgrading only for men diagnosed with Gleason 6 PC. The main outcome Kattan's tool is to determine the probability of pathologically indolent cancer in somewhat higher risk PC, thus the optimization of these two nomograms have a different focus.

## EVOLVING TECHNOLOGY FOR AS PATIENTS

The focus of this review was on clinical and pathological parameters for predicting progression of grade on AS. However, newer approaches may help in stratifying patients that have factors that place them outside the very low risk group including MRI and genomic testing. Multiparametric MRI (MPMRI) has been shown to reduce misclas-sification rates due to biopsy and has been included in the current clinical guidelines in the United Kingdom (67). Although MRI lacks sensitivity for detecting low Gleason score cancer (3+3), this might be deemed an advantage for choosing imaging negative patients for AS (68-70). However, MRI generates significant cost as well as missing roughly one-third of significant PCs (71). Incorporation of MPMRI into contemporary AS protocols will need large prospective trials to assess its impact on AS and the frequency of subsequent biopsies (72). MRI fusion is another technology that needs wider validation, but some studies have suggested it may enhance the detection of clinically relevant prostate cancers (73, 74).

Genome-based tests for detecting higher risk cancers have been a novel addition to the existing armamentarium of tools for stratifying AS patients. More widely validated tests include the Oncotype DX and Prolaris tests that utilize biopsy tissue. Oncotype DX is a 17-gene assay performed on biopsy tissue that has been shown to increase the percentage of patients choosing AS (75). Prolaris bases outcomes on the gene expression profile of 31 cell cycle progression (CCP) and 15 housekeeping genes in tissues obtained at the time of biopsy to predict aggressiveness of PC (76). Newer tests include Promark, a quantitative multiplex proteomics assay based on the expression pattern of eight protein markers to predict aggressive cancer (77). The downside of genomic testing is its significant cost ($3800 USD) potentially limiting widespread usage. In addition, estimates have suggested that the majority of risk, roughly 70%, can be determined by existing clini-copathologic parameters (78).

Finally, newer non-invasive testing may serve as an adjunct for monitoring patients on AS, including the PCA3 test. This urine-based test was analyzed in a longitudinal AS population of 240 men, but did not change longitudinally with grade reclassification (79). In another study, an incremental benefit was seen with PCA3 in an AS population for predicting Gleason >6 (80).

## CONCLUSIONS

In order to determine the probability of a patient for disease progression during conservative treatment, it is not enough to extrapolate the information from radical prostatectomy cohorts, but will be imperative to get the data from actual AS patients that progress during follow up. In order to have more accurate data, we would have to wait for the current larger cohorts to mature more, because of prostate cancer's long natural history.

Currently, the data widely supports the use of very low risk PC features for selection of patients for AS (Gleason T1c, PSA ≤10, Gleason ≤6, ≤2 positive biopsy cores, ≤50% cancer in any core, PSAD <0.15). Nomograms can be further used to stratify patients into higher risk groups for Gleason Score upgrading and subsequent reclassification during AS. Additional factors such as increased cancer volume, higher PSAD, increased body mass index, African American race or a genetic predisposition play a role in defining this increased risk. Higher risk subgroups may need a closer surveillance with more frequent biopsies (yearly), the inclusion of saturation biopsy approaches, compared to very low risk patients in which less strict protocols may be applied. The wider use of AS will ultimately benefit patients and criteria will continue to evolve as further information from longer-term AS cohorts matures and technology advances.

## Abbreviations

PC: =Prostate Cancer
AS: =Active Surveillance
GSU: =Gleason Score Upgrade
RP: =Radical Prostatectomy
PSA: =Prostate Specific Antigen
PSAD: =Prostate Specific Antigen Density
AA: =African American
